# Human DNA Helicase B Functions in Cellular Homologous Recombination and Stimulates Rad51-Mediated 5′-3′ Heteroduplex Extension *In Vitro*

**DOI:** 10.1371/journal.pone.0116852

**Published:** 2015-01-24

**Authors:** Hanjian Liu, Peijun Yan, Ellen Fanning

**Affiliations:** Department of Biological Sciences, Vanderbilt University, Nashville, Tennessee, United States of America; RIKEN Advanced Science Institute, JAPAN

## Abstract

Homologous recombination is involved in the repair of DNA damage and collapsed replication fork, and is critical for the maintenance of genomic stability. Its process involves a network of proteins with different enzymatic activities. Human DNA helicase B (HDHB) is a robust 5′-3′ DNA helicase which accumulates on chromatin in cells exposed to DNA damage. HDHB facilitates cellular recovery from replication stress, but its role in DNA damage response remains unclear. Here we report that HDHB silencing results in reduced sister chromatid exchange, impaired homologous recombination repair, and delayed RPA late-stage foci formation induced by ionizing radiation. Ectopically expressed HDHB colocalizes with Rad51, Rad52, RPA, and ssDNA. *In vitro*, HDHB stimulates Rad51-mediated heteroduplex extension in 5′-3′ direction. A helicase-defective mutant HDHB failed to promote this reaction. Our studies implicate HDHB promotes homologous recombination *in vivo* and stimulates 5′-3′ heteroduplex extension during Rad51-mediated strand exchange *in vitro*.

## Introduction

DNA double-strand break is a serious type of DNA damage because its inaccurate repair is associated with the development of cancer-prone genetic diseases. Homologous recombination is critical for the high-fidelity repair of DNA double-strand break. It is initiated by the 5′-3′ nuclease digestion of the ends of a double-strand break, resulting in 3′-single-stranded (ss) DNA [1]. The 3′-ssDNA is coated by recombinases, invades into an intact homologous duplex, and generates a displacement (D)-loop structure. The invading ssDNA extends along the homologous DNA to form a heteroduplex. Then new DNA synthesis initiates at the 3′-end of the invading ssDNA, using the homologous duplex as a template. The proteins encoded by the *Rad52* epistasis group (*Rfa1, Rad50, Mre11, Rad51, Rad52, Rad54, Rad55, Rad57, Rad59*) define this recombinational repair pathway [2]. Rad51AP1, Rad52 have been shown to stimulate the Rad51-mediated strand invasion [3], [4]. The heteroduplex extension during homologous recombination involves the unwinding of homologous DNA duplex and the extension of the invading ssDNA, and may happen in both directions. *Saccharomyces cerevisiae* Mer3 helicase has been implicated to promote the 3′-5′ heteroduplex extension to enable crossover during meiotic recombination [5]. Heteroduplex extension on the other direction (5′-3′) is more important to ensure the complete pairing of the 3′-end of ssDNA with its homologous DNA. T4 phage Dda helicase has been shown to stimulate UvsX-directed 5′-3′ heteroduplex DNA extension [6]. In eukaryotes, the ATP-dependent DNA motor protein Rad54 dissociates Rad51 from the heteroduplex and stimulates the heteroduplex extension, acting as a heteroduplex DNA pump [7], [8], [9]. Another DNA translocase Rdh54 was also shown to promote the Rad51 dissociation and D-loop formation [10]. However, whether a DNA helicase is involved in the heteroduplex extension in higher eukaryotes is not yet clear.

DNA helicase B is a robust 5′-3′ superfamily I DNA helicase conserved among vertebrates [11], [12]. Mouse and human DNA helicase B interact with DNA polymerase α-primase and stimulate the activity of DNA primase *in vitro* [12], [13]. Endogenous human DNA helicase B (HDHB) localizes in both cytoplasm and nucleus in asynchronously growing cells, a process partially regulated by CDK-dependent phosphorylation [14]. Ectopically expressed HDHB forms nuclear foci, which are stimulated by DNA damaging agents such as topoisomerase II inhibitor etoposide, or topoisomerase I inhibitor camptothecin [14].

HDHB shares sequence motifs with *E. coli* RecD and T4 Dda helicases [12]. Both of these proteins are involved in homologous recombination [1], [2]. We recently found that HDHB accumulates on chromatin in cells exposed to camptothecin, hydroxyurea, or UV [15]. Consistent with this pattern of recruitment, cells depleted of HDHB display reduced recovery from replication stress. These findings implicate HDHB is likely to play a fundamental role on the recovery of stalled or collapsed replication forks. Processing of stalled replication forks in eukaryotic cells involves Rad51-dependent homologous recombination repair [16]. This leads us to ask whether HDHB is required for homologous recombination, and if so, what role it plays.

In this study, we have further characterized the role of HDHB in DNA damage response. We show that HDHB-depleted cells have fewer sister chromatid exchange events and impaired homologous recombination repair induced by I-SceI cleavage on a chromosomal recombination reporter cassette. Ectopically expressed HDHB colocalizes with Rad51, Rad52, RPA and ssDNA. The ionizing radiation (IR)-induced RPA late-stage foci formation is reduced in HDHB-depleted cells, while Rad51 and γH2AX foci formation is not affected by HDHB silencing. Purified HDHB stimulates Rad51-mediated 5′-3′ heteroduplex extension *in vitro*. These results provide evidences that HDHB promotes cellular homologous recombination and may stimulate 5′-3′ heteroduplex extension during Rad51-mediated strand exchange.

## Materials and Methods

### Cell culture and plasmids

Human osteosarcoma U2OS cells [17] and colon carcinoma HCT116 cells [18] were grown as monolayers in Dulbecco-modified Eagle medium (DMEM) (Gibco BRL Life Technologies, Carlsbad, CA) supplemented with 10% fetal bovine serum (FBS) (Atlanta Biologicals, Norcross, GA) in a humidified 10% carbon dioxide incubator at 37°C. SW480/SN.3 cells [19] carrying a SCneo substrate [20], a kind gift from Dr. Mark Meuth, were grown in DMEM with 10% FBS and 100 μg/ml hygromycin B in a humidified 5% carbon dioxide incubator, at 37°C. pCMV5-I-SceI was a kind gift from Dr. Mark Meuth. pEGFP-C1 was purchased from Clontech (Mountain View, CA).

HCT116 cells stably expressing HDHB shRNA or control shRNA were generated as described previously [15]. The pSuper (pS) vector was kindly provided by R. Agami (The Netherlands Cancer Institute, Amsterdam, Netherlands) [21]. Two sequences targeting HDHB gene exons 1 and 2 (shRNA-1: GAGTCCGTGTTCATCGACG, shRNA-2: CAGGTGCTTGGTGGAGAGT) were selected. pS-HDHB was generated as described previously [21]. Control shRNA sequence was described previously [15]. pS-Rad51 shRNA was prepared by inserting target sequence 5′-GAGCTTGACAAACTACTTC-3′ into the pSuper vector. To examine HDHB knock-down efficiency, western blotting was performed with whole cell extracts from FACS-sorted GFP-positive cells co-transfected with pEGFP and control or HDHB shRNA.

### Clonogenic survival assay

Clonogenic survival assay was performed as described [15]. Basically, HCT116 cells were seeded in 60-mm dishes (800 cells per dish). Cells were allowed to attach to the dish for 12 h and then treated in triplicate with different concentrations of mitomycin C for 4 h, or exposed to ionizing radiation. Then cells were washed twice with PBS, and incubated in fresh DMEM for 10 days. Cell colonies were fixed and stained with 0.5% crystal violet in 70% ethanol. Visible colonies were counted.

### Micronuclei assay and sister chromatid exchange assay

HCT116 cells were grown on glass slide and irradiated by 5 Gy IR. Cells were fixed with 3.7% paraformaldehyde at room temperature for 20 min. Nuclei were stained by Hoechst 33342 (Cell Signaling, Danvers, MA).

Asynchronous HCT116 cells were grown in the presence of 15 μM BrdU in 100 mm dishes for two cell cycles (about 36 h) in the dark. Cells were treated with or without 100 ng/ml mitomycin C. To collect them, cells were trypsinized and washed once with PBS. Then they were resuspended in 10 ml pre-warmed 75 mM KCl and incubated for 10 min at 37°C. After that, cells were collected by centrifuging for 5 min at 800 rpm and resuspended in 200 μl 75 mM KCl. While gently vortexing, 5 ml pre-chilled acetic acid/methanol (1:3) was dropped into the cell suspension and mixed immediately. Fixation was performed for at least 30 min on ice. For staining, cells were collected by centrifugation, washed once with cold flesh fixative, resuspended in fresh fixative (500 μl for ∼10^7^ cells), and dropped onto wet cold slides (slides were kept in 70% ethanol at −20°C) on ice from ∼10 cm height. Slides were air-dried for 3–5 min at 50°C and stained with 10 μg/ml Hoechst 33258 (Invitrogen, Carlsbad, CA) in 10 mM phosphate buffer pH 6.8 for 20 min. Then slides were rinsed with dH_2_O, mounted with buffer (164 mM Na_2_HPO_4_ pH 7.0, 16 mM citric acid) under large-size cover slips, and exposed to long-wave UV for 1 h at 56°C. Slides were rinsed with water and immersed in 2×SSC (30 mM sodium citrate pH 7.0, 300 mM NaCl) at 60°C for 1 h. Slides were briefly dried in air, stained with 3% Giemsa in 10 mM phosphate buffer for 12 min, mounted and observed with microscope. 100 cells in three experiments were counted.

### *In vivo* recombination assay

1.2×10^6^ SW480/SN.3 cells were replated onto a 60 mm dish. 24 h later, cells were transiently transfected with 6 μg pS-control or pS-HDHB-shRNA together with 2 μg pCMV5-I-SceI in Lipofectamine 2000 (Invitrogen, Calsbad, CA). pFLAG was used as a control vector for I-SceI. Cells were grown in DMEM for 48 h, with one change of fresh DMEM medium at 24 h after transfection. Then cells were trypsinized and replated in triplicate into 100 mm dishes with fresh DMEM. To measure the plating efficiency, about 800 cells were plated in dishes without G418 (Gibco BRL Life Technologies, Carlsbad, CA). To select neo-resistant cells, 1×10^6^ cells were replated into a dish supplemented with 1 mg/ml G418 in the medium. Colonies formed after growth for 11–12 days were stained with 0.5% crystal violet in 70% ethanol.

To verify the recombination products in cells, single colony was picked and expanded. Genomic DNA was extracted with DNAeasy kit (QIAGEN, Valencia, CA). PCR amplification was performed by using two primers: CGAGCAGTGTGGTTTTGCAAGAGG and GTCAAGAAGGCGATAGAAGGCGATG against the recombination substrate on the genomic DNA. PCR products were purified by QIAquick PCR purification kit (QIAGEN, Valencia, CA) and cut with NcoI. The digested products were electrophoresed through 2% agarose gel in 0.5×TBE buffer and visualized by ethidium bromide staining.

HDHB silent mutations which confer resistance to shRNA-1 were generated by site-directed mutagenesis. Three silent third-codon mutations (112-GAATCGGTATTC-123) were introduced to target sequence (112-GAGTCCGTGTCC-123). The full-length mutant HDHB sequence was inserted into a pRetroX-TetOne-Puro vector (Clontech, Mountain View, CA). The constructed plasmid was then transfected into the retrovirus packaging cell line GP2-293 with envelop vector VSV-G. Retroviral supernatant was harvested 48 hours after transfection. SW480/SN.3 cells were infected with the viral supernatant supplemented with 4 μg/ml polybrene (Sigma-Aldrich). After infecting for 24 hours, cells were selected by 5 μg/ml puromycin (Sigma-Aldrich). To induce the expression of silent mutant HDHB, different concentrations of doxycycline were added to cell culture for 48 hours. Then doxycycline was removed.

### Flow cytometry and cell sorting

For flow cytometric analysis, cells were collected by trypsin treatment, washed with PBS+2%FBS, and fixed in 70% ethanol for 1 h at 4°C. After a PBS wash, cells were incubated for 30 min with 10 μg/ml propidium iodide and 250 μU/ml RNase A (Calbiochem, Billerica, MA) at 37°C. Cell cycle analysis was done on a FACScan (BD Biosciences, San Diego, CA) at Vanderbilt Flow Cytometry Services Facility.

To sort GFP-positive cells, cells were co-transfected with pEGFP-C1 (Clontech, Mountain View, CA) and HDHB shRNA, and sorted on a FACSAria (BD Biosciences, San Diego, CA) at Vanderbilt Flow Cytometry Services Facility.

### Fluorescence microscopy

GFP-HDHB was transiently expressed in U2OS cells as described previously [14]. To perform cell imaging, cells were replated on cover slips in 35 mm dishes and treated with 5 Gy IR 24 hours later. At the indicated time points after IR, cells were extracted for 5 min on ice with 0.2% Triton X-100 in CSK buffer (10 mM HEPES, pH 7.4, 300 mM sucrose, 100 mM NaCl, 3 mM MgCl_2_, supplemented with 1×protease inhibitors) and then fixed with 3.7% paraformaldehyde at room temperature for 20 min. Cells were stained with anti-RPA34 antibody (1:500 dilution in phosphate-buffered saline (PBS)) supplemented with 10% FBS at room temperature for 2 hours. After washing with PBS, cells were incubated with Cy3-conjugated secondary antibody (Jackson ImmunoResearch Laboratories, West Grove, PA) (1:100 dilution) for 1 h at room temperature. After three washes, cells were incubated for 10 min with To-Pro-3 iodide (Invitrogen, Calsbad, CA) at a concentration of 3 μM in PBS. Coverslips were mounted in ProLong Antifade (Molecular Probes, Eugene, OR). Fluorescence pictures were taken on an Axioplan 2 imaging system as described [14]. Anti-Rad52 and monoclonal anti-Rad51 primary antibodies were purchased from Novus (Littleton, CO) and diluted 1:300 in PBS with 10% FBS. Rabbit anti-γH2AX phospho-Ser139 antibody was purchased from Upstate (Charlottesville, VA) and diluted 1:500 in PBS with 10% FBS. BrdU monoclonal antibody was purchased from Becton Dickinson (Franklin Lakes, NJ).

### DNA substrates

X174 circular ssDNA and RFI dsDNA were purchased from NEB (Ipswich, MA). Linear X174 dsDNAs with different types of termini were prepared by cleaving X174 RFI dsDNA with restriction endonucleases. Linear dsDNA with two blunt ends was made by digestion with SspI endonuclease. Linear dsDNA with 3′-overhang was made with PstI digestion. Linear dsDNA with 5′-overhang was made with XhoI digestion. The digested DNA was purified with a QIAquick PCR purification kit (QIAGEN, Valencia, CA). Linear dsDNA with a blunt end and a 3′-overhanging terminus was created with a double digestion of PstI and StuI. Linear dsDNA with a blunt end and a 5′-overhanging terminus was created with a double digestion of XhoI and SspI. Linear dsDNA with a blunt end and a 3′-recessive terminus was made by double digestion with XhoI and StuI. Linear dsDNA with a blunt end and a 5′-recessed terminus was made by double digestion with PstI and SspI. After double digestions, the products were separated by 1% agarose gel electrophoresis and the large DNA fragment was purified with the QIAquick gel extraction kit (QIAGEN, Valencia, CA). To label the linear dsDNA with 3′-overhanging termini, PstI-digested X174 dsDNA was firstly dephosphorylated with Antarctic phosphatase (NEB, Ipswich, MA). To get more efficient labeling of the 5′-recessed end, DNA was heated at 70°C for 10 min, chilled on ice, and then labeled with T4 polynucleotide kinase (NEB, Ipswich, MA) and [γ^32^-P]-ATP (Amersham Biosciences, UK). The labeled dsDNA was purified with a Sephadex G-50 column (Roche). To prepare the nicked-circular DNA marker, 0.5 μg linear dsDNA and 1.5 μg circular ssDNA were denatured and annealed in 20 μl annealing buffer (20 mM Tris-HCl pH 8.0, 50 mM NaCl, 10 mM MgCl_2_).

### Proteins

*E. coli* vector pET15b-Rad51-2 expressing wild-type full-length 6×His-Rad51 was a kind gift from Dr. Walter Chazin. The purification of Rad51 was performed as described [22]. Recombinant human RPA heterotrimer was expressed and prepared as described [23]. RPA was diluted in dialysis buffer (20 mM Tris-HCl pH 7.8, 50 mM NaCl, 10 μM ZnCl_2_, 5% glycerol, 1 mM DTT) to about 1 mg/ml to reduce protein loss during dialysis and dialyzed overnight. RPA concentration was determined with the Bradford assay (Bio-Rad). Recombinant wild-type and mutant HDHB were expressed and prepared as described [12].

### Strand exchange assay

Rad51-mediated strand exchange reactions (20 μl) were performed in reaction buffer (40 mM Tris-HCl pH7.5, 1 mM MgCl_2_, 1 mM DTT, 2.5 mM ATP, 8 mM creatine phosphate, 28 μg/ml creatine kinase) supplemented with different salts as indicated in the figure legends. Rad51 (7.5 μM) was mixed with X174 circular ssDNA (30 μM as nucleotides) at 37°C for 5 min, then RPA (1.5 μM) was added and the incubation was continued for another 5 min. After that, different amounts of HDHB (50 nM, 100 nM, or 150 nM) were added into the reaction. For Walker B mutant HDHB, the concentration was 100 nM. Strand exchange was initiated by adding linearized X174 dsDNA (30 μM as nucleotides) to the reaction. For reactions performed with (NH_4_)_2_SO_4_ as the salt, (NH_4_)_2_SO_4_ was added between the additions of RPA and HDHB. Aliquots were withdrawn from the reaction at indicated times, and deproteinized by adding SDS to 0.5% and proteinase K to 1 mg/ml, followed by incubation at 37°C for 20 min. Reaction products were mixed with 6×loading dye (30% glycerol, 0.25% bromophenol blue, 0.25% xylene cyanol) and separated by electrophoresis in a 0.9% agarose gel in 1×TAE buffer. Both the gel and the running buffer were supplemented with 1 μg/ml ethidium bromide. The electrophoresis was performed at 2 V/cm for about 3 h. The gel was destained in ample dH_2_O for 4 h and visualized under short-wave UV. Quantification of reaction products was conducted by densitometric scanning, using IPLabgel 1.5 (Signal Analytics Corp.), of the autoradiograph film.

## Results

### HDHB-depleted cells have reduced sister chromatid exchange rate

**Figure 1:**
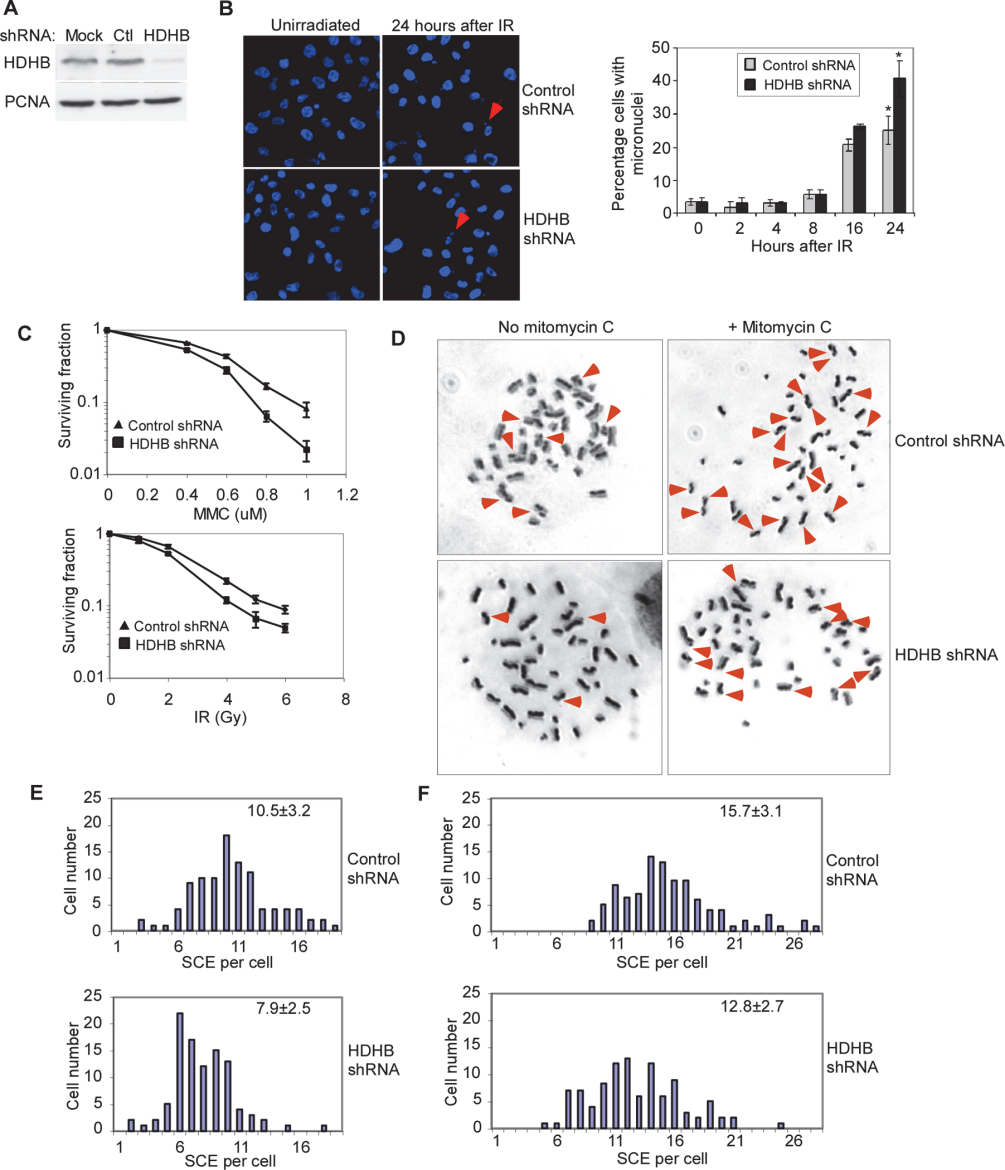
HDHB-depleted cells have impaired DNA repair and reduced sister chromatid exchange rate. (A) Western blotting of HCT116 cells expressing short hairpin RNA (shRNA) against control (Ctl) or HDHB. (B) Left, example of micronuclei in cells exposed to 5 Gy ionizing radiation (IR). Right, percentage of cells with micronuclei. 300 cells from three independent experiments were counted. Error bars represent mean ± s.d. *P≤0.05, Student *t*-test. Arrows indicate micronuclei. (C) Colonies formed by surviving HCT116 cells treated with mitomycin C (MMC), or IR. Untreated cells were counted as 100% survival. The mean values ± s.d. from three independent experiments are plotted. (D) Sister chromatid exchange (SCE) of HCT116 cells expressing HDHB shRNA or control shRNA treated without mitomycin C (left) or with 100 ng/ml mitomycin C (right). Arrows indicate the exchange events. (E), (F) Analysis of SCE events in 100 cells expressing HDHB shRNA or control shRNA treated without mitomycin C (E) or with 100 ng/ml mitomycin C (F). The value at the top right of each panel is the mean ± s.d. SCE per cell from three independent experiments.

We previously showed that HDHB-depleted cells were more sensitive to camptothecin-induced DNA damage [15]. To further assess the importance of HDHB in DNA damage response, endogenous HDHB was stably depleted from HCT116 cells with small hairpin RNA (shRNA) (Fig. 1A). Equal numbers of HDHB-depleted cells and control cells were treated with mitomycin C, or IR. HDHB-depleted cells showed more micronuclei after IR than control cells (Fig. 1B), suggesting that they were defective to maintain chromosome stability after IR. The ability of HDHB-depleted HCT116 cells to survive and form colonies after IR exposure was reproducibly reduced to about half that of cells expressing control shRNA (Fig. 1C). Moreover, HDHB-depleted cells were significantly more sensitive to mitomycin C than cells expressing control shRNA (Fig. 1C). Homologous recombination repair or non-homologous end joining are both involved in the repair of IR induced double-strand breaks [24]. Homologous recombination repair plays a primarily role in the repair of mitomycin C- and camptothecin-induced DNA damages [25], [26], [27]. Our findings that HDHB-depleted cells were sensitive to IR, mitomycin C and camptothecin are consistent with a potential function of HDHB in homologous recombination repair.

Homologous recombination has been shown to mediate sister chromatid exchange [28]. To test sister chromatid exchange rate, we treated HDHB-depleted HCT116 cells and cells expressing control shRNA with or without mitomycin C, and differentially stained the two sister chromatids in metaphase-arrested cells (Fig. 1D). The frequency of spontaneous sister chromatid exchange events was lower in HDHB-depleted cells than in control cells in the absence of DNA damage treatment (Fig. 1E). Student *t*-test showed the difference between the two cells was statistically significant at the 0.05 level. Treatment with 100 ng/ml mitomycin C increased sister chromatid exchange rate in both cells. However, HDHB-depleted cells again showed fewer sister chromatid exchanges per cell than cells expressing control shRNA after exposure to mitomycin C (Fig. 1F). These findings provide initial evidence that HDHB may function in homologous recombination in the absence of exogenous damage, as well as after exposure to DNA damaging agents.

### HDHB Facilitates homologous recombination repair *in vivo*

**Figure 2:**
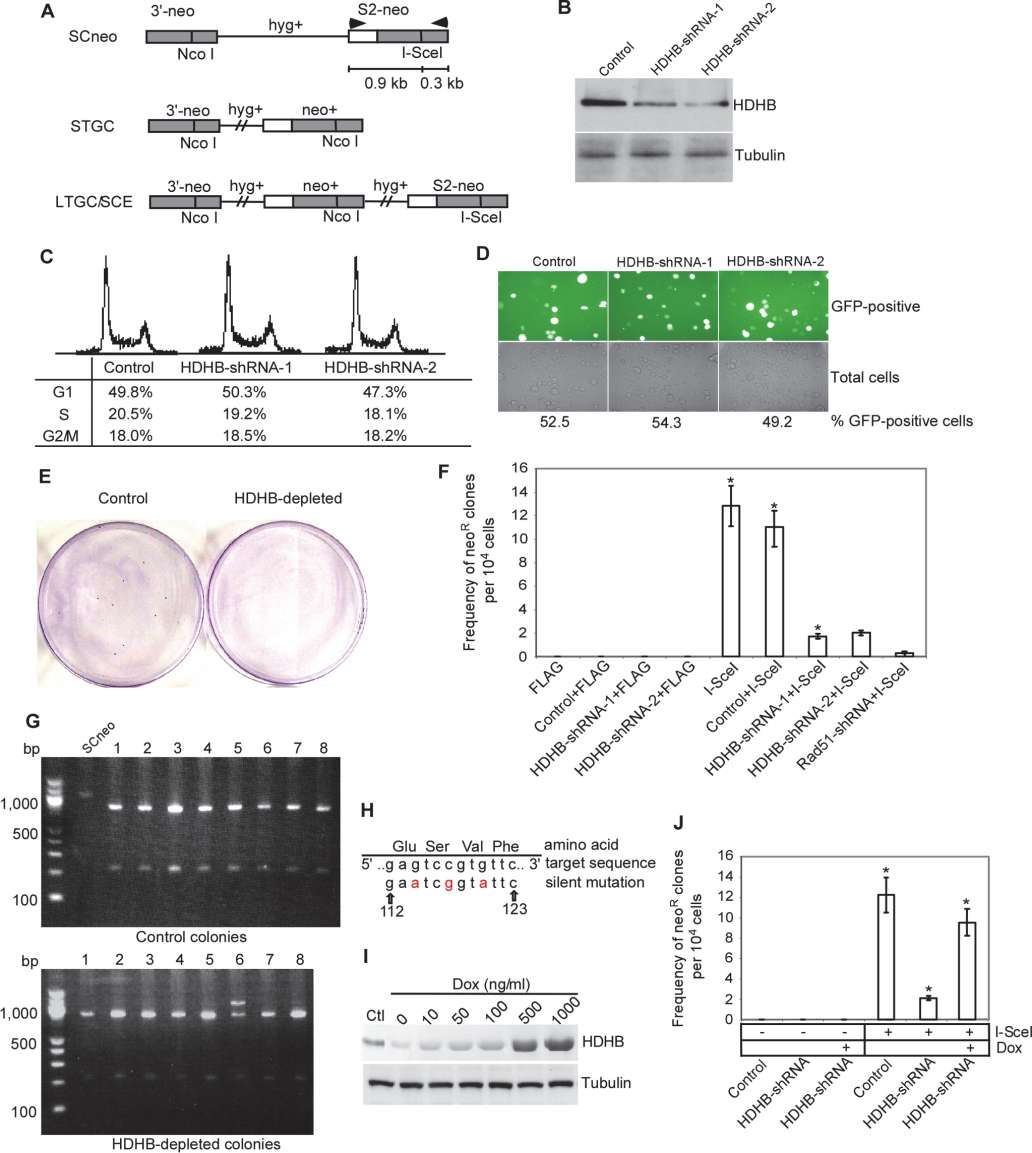
HDHB depletion impairs homologous recombination repair *in vivo*. (A) Schematic of *in vivo* recombination assay. I-SceI-induced double-strand break can be repaired by homologous recombination and result in short tract gene conversion (STGC) or long tract gene conversion (LTGC)/sister chromatid exchange (SCE). Arrows indicate the target positions of PCR primers used in Fig. 2G. (B) Western blotting of cell extracts with or without HDHB silencing. Cells were co-transfected with pEGFP and sorted by FACS. Quantification of reaction products was conducted by densitometric scanning, using IPLabgel 1.5. HDHB knockdown efficiencies were 75% for HDHB-shRNA-1 and 80% for HDHB-shRNA-2. (C) Cell cycle analysis of SW480 cells transfected with control shRNA or HDHB shRNA. (D) Transfection efficiency of control shRNA and HDHB shRNA into SW480 cells evaluated by GFP co-transfection and expression. (E) G418-resistant colonies formed after co-transfection of I-SceI with control shRNA or HDHB shRNA. (F) Frequency of neo-resistant colonies per 10^4^ cells. pFLAG was used as a control vector for I-SceI. The mean values ± s.d. from three independent experiments are plotted. *P<0.01, Student *t*-test. (G) PCR products amplified from the genomic DNA of surviving colonies were cut with NcoI and analyzed by agarose-gel electrophoresis. Lane 6 of the lower panel indicates a long tract gene conversion or sister chromatid exchange (LTGC/SCE). (H) Three silent third-codon HDHB mutations which confer resistance to shRNA-1. Red letters are the mutant nucleotides. (I) Western blotting of whole cell extracts of SW480/SN.3 cells. SW480/SN.3 cells bearing doxycycline-inducible silent mutant HDHB were transfected with HDHB shRNA-1. To induce the expression of silent mutant HDHB, different concentrations of doxycycline (Dox) were added to cell culture for 48 hours. Ctl, control cells without shRNA-1 transfection and doxycycline treatment. (J) Frequency of neo-resistant colonies per 10^4^ cells. SW480/SN.3 cells bearing doxycycline-inducible silent mutant HDHB were transfected with HDHB shRNA-1 or control shRNA, together with I-SceI expression plasmids, and then treated with or without 50 ng/ml doxycycline. The mean values ± s.d. from three independent experiments are plotted. *P<0.01, Student *t*-test. Doxycyline treatment rescued the reduced homologous recombination in HDHB shRNA transfected cells.

To further test the potential role of HDHB in homologous recombination repair, we performed an *in vivo* recombination assay developed by Jasin and colleagues [20]. SW480/SN.3 cells bearing an integrated SCneo recombination reporter cassette were used [17]. This SCneo reporter cassette contains a neomycin-resistant gene disrupted by an I-SceI restriction site. An intact copy of the disrupted portion of the neomycin gene is present at the other end of the cassette (Fig. 2A). If the double-strand break created by I-SceI expression is repaired by homologous recombination, an intact neomycin-resistant gene will be generated and the cells that have undergone successful homologous recombination repair can be identified by selecting for growth in G418.

We co-transfected SW480/SN.3 cells with I-SceI expression vector and vectors encoding two different shRNAs against HDHB or a control sequence. Co-transfection with HDHB shRNA showed no effect on cell cycle distribution or transfection efficiency (Fig. 2C and 2D). Western blotting showed 75–80% HDHB knock-down efficiency in successfully transfected cells (Fig. 2B). Transfection of HDHB shRNA into the cells appeared to have no significant influence on cell viability or growth, as similar numbers of colonies from cells transfected with HDHB shRNA or with control vector were obtained after growth in medium without G418 (data not shown). Transfection of I-SceI into the cells promoted the formation of G418-resistant colonies (Fig. 2E). However, HDHB shRNA transfected cells showed a significant reduction in G418-resistant colony formation compared to control shRNA transfected cells (Fig. 2E and 2F). Statistical significance by Student’s *t*-test is P≤0.01. Co-transfecting I-SceI with Rad51 shRNA showed even greater reduction in G418-resistant colony formation. Recombination of the neomycin-resistance gene at the I-SceI site in G418-resistant cells were confirmed by amplifying a region of the recombination substrate cassette using PCR and cutting with NcoI (Fig. 2G). To confirm the reduction of homologous recombination rate is due to the depletion of HDHB, we generated a SW480/SN.3 cell line expressing inducible silent mutant HDHB (Fig. 2H). Cells treated with 50 ng/ml doxycycline can express silent mutant HDHB (Fig. 2I) and grow well. We co-transfected these cells with I-SceI expression vector and HDHB shRNA. Then we treated them with 50 ng/ml doxycyline. Doxycycline treatment rescued the reduced homologous recombination in HDHB shRNA transfected cells (Fig. 2J). These results indicate that the depletion of HDHB impairs homologous recombination repair in cells.

### RPA late-stage foci formation after ionizing radiation is delayed in HDHB-depleted cells

**Figure 3:**
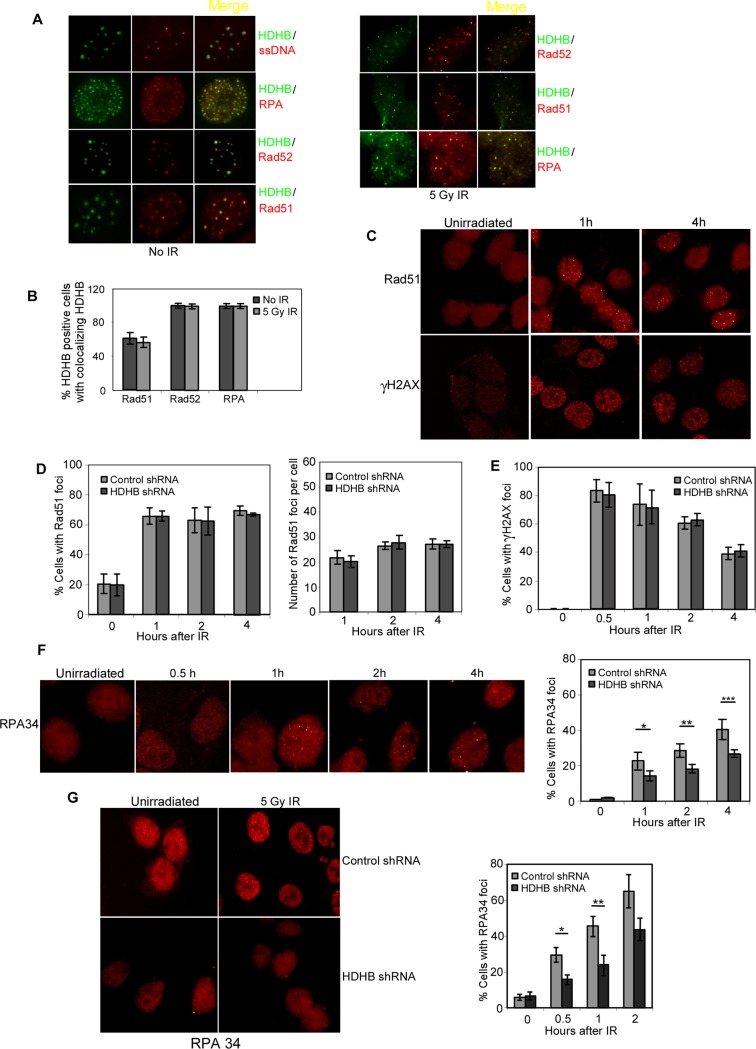
HDHB depletion impairs RPA late-stage foci formation after IR. (A) U2OS cells transiently expressing GFP-HDHB were stained with different antibodies and observed by immunofluorescence. To visualize ssDNA, cells were grown in BrdU for 24 hours, fixed and stained with anti-BrdU antibody. Left, no IR; Right, cells were fixed at 2 h after 5 Gy IR treatment. (B) Percentage of HDHB-positive cells with Rad51, Rad52 or RPA foci colocalizing with HDHB. 500 cells in three experiments were counted. The mean values ± s.d. are plotted. (C) Example of 5 Gy IR-induced Rad51 and γH2AX foci in HCT116 cells at 1 h or 4 h after irradiation. (D) Percentage of cells with Rad51 foci after IR. Right panel is the mean number of Rad51 foci per cell after IR. Total 1000 cells in three experiments were counted. The mean values ± s.d. are plotted. (E) Percentage of cells with γH2AX foci after IR. (F) Left, example of RPA34 foci in HCT116 cells at 0.5 h, 1 h, 2 h, 4 h after 5 Gy IR. Right, percentage of cells with RPA34 foci after IR. *P<0.05, **P<0.05, ***P<0.05, Student *t*-test. (G) Left, example of RPA34 foci in U2OS cells transfected with control shRNA or HDHB shRNA at one hour after 5 Gy IR. Right, percentage of cells with RPA34 foci after 5 Gy IR at different time points. *P<0.05, **P<0.05, Student t-test.

Rad51 and RPA form foci after ionizing radiation, which co-localize with γH2AX foci, an indicator of double-strand breaks [29], [30]. Ectopically expressed HDHB localizes in nuclear foci induced by DNA damaging agents [14]. To see whether these foci are related to homologous recombination repair, we performed immunofluorescence with anti-Rad51, Rad52, and RPA antibodies in cells. In a significant portion of cells, GFP-HDHB colocalized with ssDNA, RPA, Rad52, and Rad51 in the presence or absence of irradiation (Fig. 3A and 3B).

RPA is thought to have two roles in homologous recombination-mediated repair of double-strand breaks, first in facilitating formation of the recombinogenic Rad51-ssDNA filament, and later in stabilizing the displaced ssDNA after Rad51-mediated strand invasion of a homologous duplex DNA [31], [32], [33]. To better understand how HDHB participates in homologous recombination repair, we tested IR-induced RPA foci formation in HCT116 cells that were expressing HDHB shRNA or control shRNA. After irradiation, small bright foci of Rad51 and γH2AX formed within 1 h (Fig. 3C). There was no significant difference between cells expressing HDHB shRNA or control shRNA in the percentage of cells displaying Rad51 or γH2AX foci in the first few hours (Fig. 3D and 3E). RPA34 formed two types of foci. Small RPA34 foci formed quickly in 0.5 h after irradiation. Large bright RPA34 foci appeared more slowly than Rad51 foci during the first 4 h after IR (Fig. 3F). The fraction of cells displaying IR-induced late-stage RPA foci was greater for cells expressing control shRNA than for cells expressing HDHB shRNA (Fig. 3F). Similar results were observed from experiments in U2OS cells transiently transfected with HDHB shRNA (Fig. 3G).

The observation that early γH2AX and Rad51 foci formed normally in HDHB-depleted cells thus suggests that the dissection of double-strand break ends and the loading of Rad51 during homologous recombination repair are not dependent on HDHB. On the other hand, the slower formation of late-stage RPA foci in HDHB-depleted cells implicates a defect in the formation or stabilization of the displaced ssDNA during strand exchange, which would be coated by RPA and visible as RPA late-stage foci. Considering the formation of the displaced ssDNA is driven by the extension of the invading ssDNA along the homologous DNA duplex, this result leads us to ask whether HDHB is involved in the heteroduplex extension reaction during homologous recombination.

### HDHB stimulates Rad51-mediated 5′-3′ heteroduplex extension

**Figure 4:**
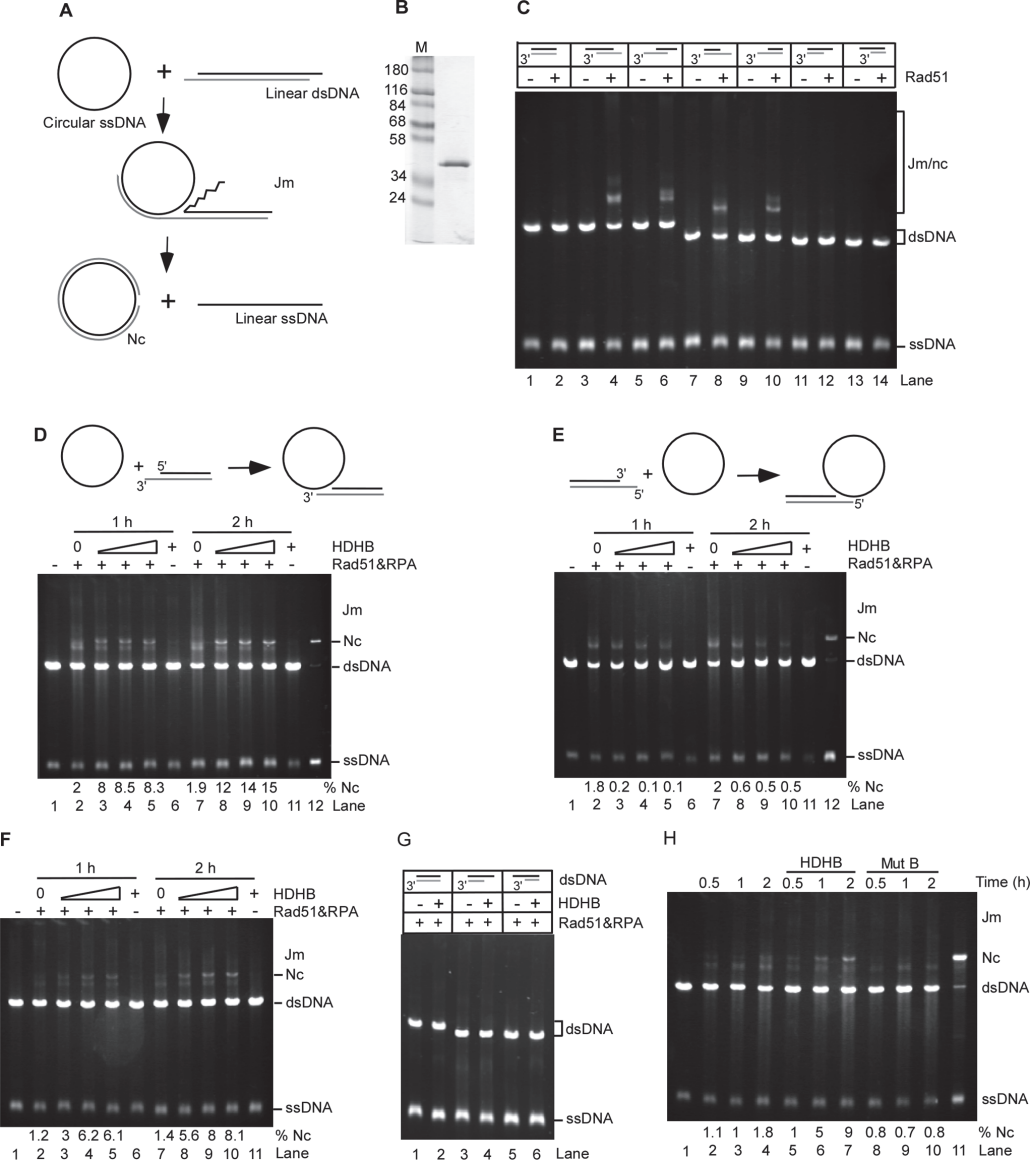
HDHB stimulates 5′-3′ Rad51-mediated heteroduplex extension. (A) Diagram of strand exchange reaction. Jm, joint molecule; nc, nicked-circular DNA. (B) hRad51 purification. (C) DNA-end requirements for hRad51-catalyzed strand exchange. Different linear dsDNA substrates as indicated were used in the strand exchange reactions supplied with 100 mM (NH_4_)_2_SO_4_. The gray strand of each dsDNA substrate is the strand complementary to the circular ssDNA. The reactions were stopped 2 h after the reactions were initiated. (D), (E) Strand exchange reactions were performed in the presence of 60 mM KCl and 2 mM CaCl_2_. dsDNA with 3′-overhanging termini (D) or 5′-overhanging termini (E) was used in the reaction. An annealed nicked-circular DNA marker is in lane 12. HDHB concentration in the reactions was: lanes 3 and 8, 50 nM; lanes 4 and 9, 100 nM; lanes 5 and 10, 150 nM. Quantification of nicked-circular DNA formed in the reaction was shown on the bottom of each gel. (F) Strand exchange reactions were performed in the presence of 50 mM (NH_4_)_2_SO_4_. dsDNA with 3′-overhanging termini was used. (G) Reaction was performed with dsDNA with blunt-end termini or recessed end. HDHB concentration was 100 nM. (H) Walker B mutant HDHB did not promote heteroduplex extension. The concentration of wild-type or mutant HDHB was 100 nM.

We investigated Rad51-mediated strand exchange in the presence of HDHB. Human Rad51 (hRad51) was purified as described (Fig. 4B) [34]. An *in vitro* strand-exchange reaction between linear X174 dsDNA and circular ssDNA catalyzed by hRad51 was performed (Fig. 4A) in the presence of either (NH_4_)_2_SO_4_ or CaCl_2_ [35], [36], [37], [38]. We firstly tested the end requirements for DNA substrates in the strand-exchange reaction. Consistent with previous findings in yeast [39], [40], [41], hRad51 only promoted strand exchange in the reactions containing dsDNAs with at least one overhanging ssDNA tail on the complementary strand (Fig. 4C).

The hRad51-catalyzed strand-exchange reaction was then examined in the presence of HDHB. Reactions were performed in the presence of 60 mM KCl and 2 mM CaCl_2_, or 50 mM (NH_4_)_2_SO_4_ (Fig. 4D, 4E and 4F). HDHB stimulated the formation of nicked-circular DNA, a completely exchanged product, when the reaction was initiated from the 5′-end with respect to the displaced ssDNA (Fig. 4D). The formation of nicked-circular DNA required Rad51 and RPA (Fig. 4D and 4E, lanes 6, 11). On the other hand, the formation of both nicked-circular DNA and joint molecules was inhibited by HDHB when the reaction started from the 3′-end of the displaced ssDNA (Fig. 4E). This could result from the disruption of nascent joint molecules by the 5′-3′ translocation of HDHB on the circular ssDNA. The 5′-3′ stimulation is correlated with HDHB concentration (Fig. 4F, lanes 3 and 4, lanes 8 and 9). HDHB did not promote the formation of pairing products for linear dsDNA with blunt ends or linear dsDNA with recessed ends of the complementary strand (Fig. 4G).

A helicase-deficient Walker-B mutant HDHB [12] failed to stimulate the formation of nicked-circular DNA, indicating that HDHB helicase activity is required for the promotion (Fig. 4H). .

**Figure 5:**
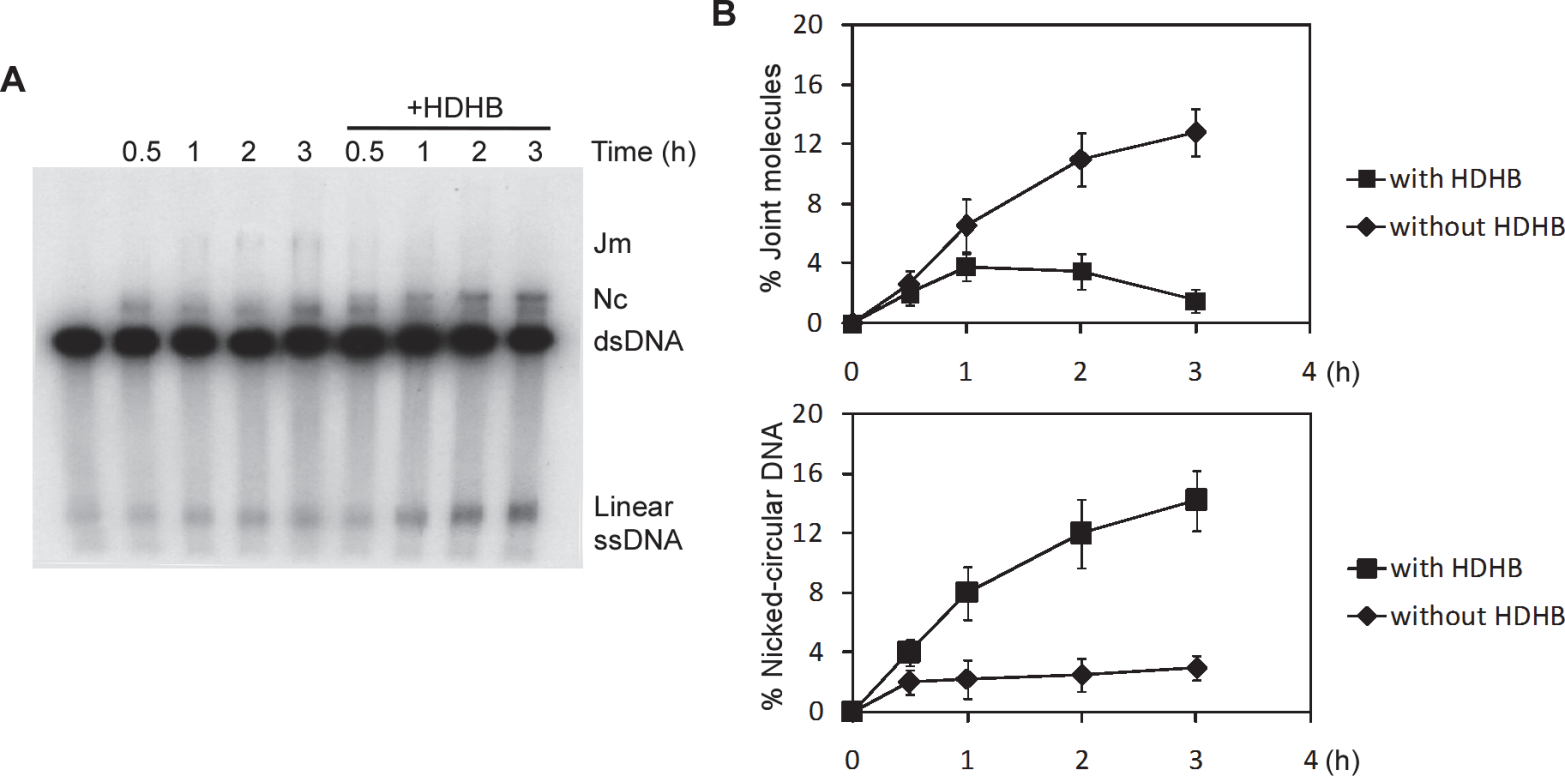
HDHB promotes heteroduplex extension but not joint molecule formation. (A) Strand exchange reaction was performed with ^32^P-labeled linear dsDNA and in the presence of 50 mM (NH_4_)_2_SO_4_. The concentration of HDHB was 100 nM. (B) Quantitative analysis of jm and nc DNA as the percentage of initial dsDNA. The mean values ± s.d. from three independent experiments are plotted.

To better understand the mechanism of the stimulation, we labeled the 5′-end of the linear dsDNA with ^32^P and quantified the reaction products. Rad51 promoted the formation of joint molecules gradually in 3 h (Fig. 5). In the presence of HDHB, the formation of nicked circular DNA was significantly stimulated. This was accompanied by the releasing of linear ssDNA (Fig. 5A). The amount of joint molecules and nicked-circular DNA formed in the reactions was quantified. Although more nicked-circular DNA was formed in the presence of HDHB than in its absence, the amount of joint molecules decreased in parallel (Fig. 5B). The total amount of reaction products (joint molecules and nicked circular DNA) was not increased in HDHB-containing reactions as compared to control reaction, suggesting that HDHB stimulates nicked circular DNA formation by promoting the heteroduplex extension, but not the formation of joint molecules.

## Discussion

Previous study showed that GFP-HDHB forms foci in both G1 and S/G2 cells [14]. During G1, HDHB primarily localizes in nucleus. Although the function of HDHB in G1 remains to be determined, further study showed that UV, hydroxyurea, and camptothecin induce accumulation of HDHB on chromatin in S phase cells [15]. Moreover, cells depleted of HDHB display reduced recovery from replication stress [15]. These findings implicate HDHB is likely to have a fundamental role on the recovery of stalled or collapsed replication forks during S phase. We describe here that HDHB silencing results in hyper-sensitivity to mitomycin C and IR, reduced sister chromatid exchange and impaired homologous recombination repair *in vivo*. These results are consistent with the interpretation that depletion of HDHB reduces homologous recombination-dependent double-strand break repair. We note that depletion of Rad51 showed even larger inhibition of homologous recombination repair than depletion of HDHB, thus HDHB may promote but not be absolutely required for homologous recombination repair.

GFP-HDHB colocalized with Rad52, Rad51, RPA and ssDNA. We show that the early formation of γH2AX and Rad51 foci after IR was not affected by HDHB depletion. However, the formation of RPA late-stage foci after IR was impaired and/or retarded in HDHB-depleted cells. In interpreting this observation, it is important to consider that RPA has at least two roles in homologous recombination repair, first in facilitating the assembly of the Rad51-ssDNA filament prior to the invasion of the donor duplex and later in stabilizing the displaced ssDNA after Rad51-mediated strand invasion [31], [32], [33]. The observation that early γH2AX and Rad51 foci formed normally in HDHB-depleted cells thus suggests that the processing of double-strand break ends and the loading of Rad51 during homologous recombination repair are not dependent on HDHB. In contrast, the slow formation of large RPA foci in HDHB-depleted cells suggests a defect in the formation or stabilization of the displaced ssDNA during strand exchange, which would be coated by RPA and visible as RPA late-stage foci. This result implies the extension of invading ssDNA along the homologous DNA duplex is impaired in HDHB-depleted cells.

Consistent with this interpretation, HDHB stimulated the formation of completely exchanged products in a Rad51-mediated 5′-3′ strand exchange reaction *in vitro*. The strand exchange in the reverse direction was inhibited by HDHB. Furthermore, HDHB did not promote the formation of joint molecules, but only the heteroduplex extension step. Because HDHB is a 5′-3′ DNA helicase [12], our results are consistent with HDHB translocating 5′-3′ on either the displaced ssDNA strand or the circular ssDNA, resulting in 5′-3′ promotion of heteroduplex extension or 3′-5′ inhibition of strand pairing. Considering the tailed duplex DNA is the preferred substrate for Rad51 protein-mediated homologous pairing [42], strand invasion is more likely to initiate from the 5′-end of the invading ssDNA. The 5′-3′ extension direction can ensure the 3′-end of the invading ssDNA completely pair with the complementary strand.

**Figure 6:**
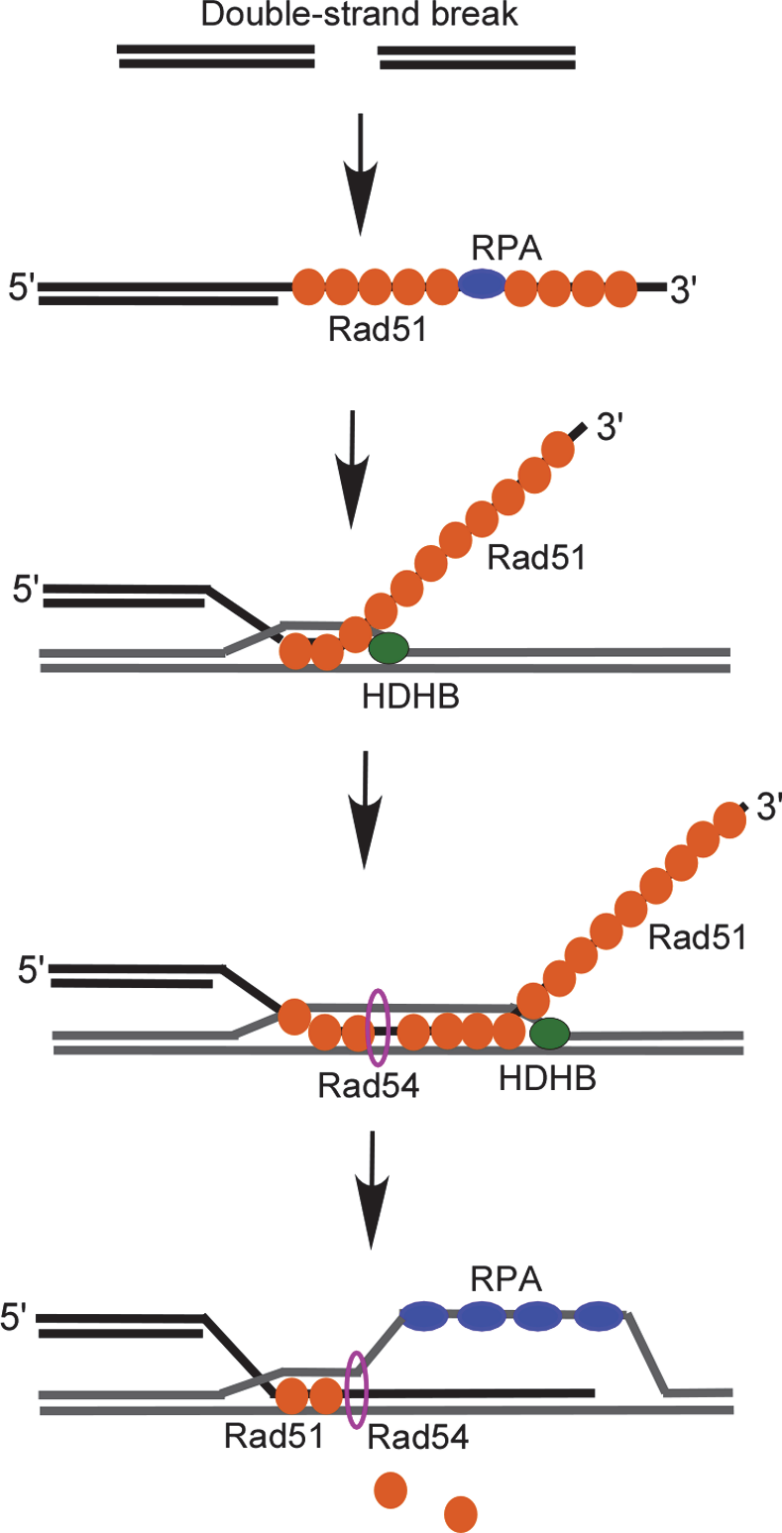
Model depicting how HDHB may function in heteroduplex extension. During homologous recombination *in vivo*, the invading strand is coated with Rad51. The 5′-end of the invading ssDNA, which has a duplex DNA tail, would preferentially invade the homologous duplex. HDHB may translocate 5′-3′ along ssDNA and accelerate the 5′-3′ heteroduplex extension after strand invasion.

Rad54 is a motor protein to function in many different steps during homologous recombination [43]. It can stimulate Rad51-mediated strand invasion, chromatin remodeling, and the postsynaptic DNA extension. Rad54 is a dsDNA-dependent ATPase, but it doesn’t have a helicase function. Heteroduplex DNA extension involves unwinding of the double-stranded DNA. It will be interesting to see whether HDHB functions with Rad54 to accelerate the formation of D loop in the future (Fig. 6).
