# Correction: Human DNA Helicase B Functions in Cellular Homologous Recombination and Stimulates Rad51-Mediated 5′-3′ Heteroduplex Extension *In Vitro*

**DOI:** 10.1371/journal.pone.0205766

**Published:** 2018-10-10

**Authors:** The PLOS ONE Editors

Following publication of this article [1], it was brought to the attention of the *PLOS ONE* Editors that author Ellen Fanning was deceased at the time of submission, and therefore could not approve the final version of the manuscript or agree to the submission of the manuscript to *PLOS ONE*. This was not declared. A corrected author list is as follows:

Hanjian Liu, Peijun Yan, Ellen Fanning†

†Deceased September 1, 2013

Furthermore, the content of the article is based on a chapter of the doctoral thesis of corresponding author Hanjian Liu, which was supervised by Ellen Fanning at Vanderbilt University. Follow-up experiments to complete the published study were performed by corresponding author Hanjian Liu using facilities at the University of Science and Technology of China.

The *PLOS* Data Availability policy requires authors to make all data underlying the study findings available. The Data Availability statement for this article indicates that all relevant data are within the paper; however, the individual-level data points underlying the charts and a number of the original, uncropped image files are not included with the article. The corresponding author has provided the underlying dataset, which is included here as S1 File; however, a number of original files are no longer available. The original uncropped image files or data sets underlying the following figures have not been provided:

Fig 1A Western blots, Fig 1E top panel chart, Fig 1F charts, Fig 2B Western blots, Fig 2C flow cytometry cell cycle analysis, Fig 2D image, Fig 2E image, Fig 2I Western blot, Fig 3A immunofluorescent microscopy images.

## Supporting information

S1 File Supplementary dataset. This file includes the underlying dataset.
(ZIP)
