# Supplementary figures and images for: Correction: Human DNA Helicase B Functions in Cellular Homologous Recombination and Stimulates Rad51-Mediated 5′-3′ Heteroduplex Extension In Vitro
Source: PLoS One. 2018 Oct 10;13(10):e0205766. doi: 10.1371/journal.pone.0205766 (PMC6179301; doi:10.1371/journal.pone.0205766)

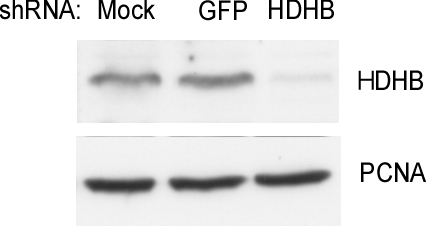

Supplement: S1 File — This file includes the underlying dataset. (ZIP) [file pone.0205766.s001.zip › fig 1A.jpg]

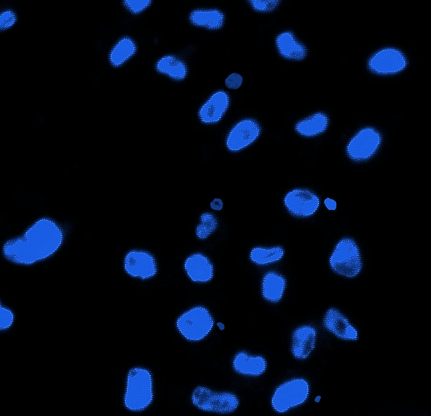

Supplement: S1 File — This file includes the underlying dataset. (ZIP) [file pone.0205766.s001.zip › Fig 1B bottom right panel image1.tif]

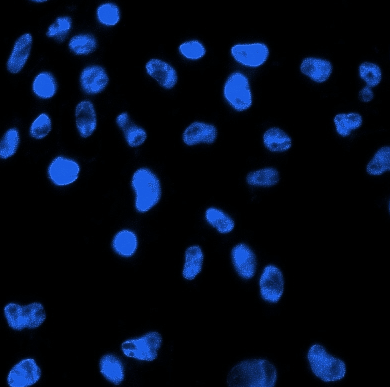

Supplement: S1 File — This file includes the underlying dataset. (ZIP) [file pone.0205766.s001.zip › Fig 1B left bottom.tif]

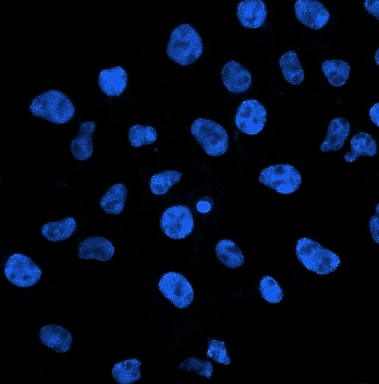

Supplement: S1 File — This file includes the underlying dataset. (ZIP) [file pone.0205766.s001.zip › Fig 1B left top.tif]

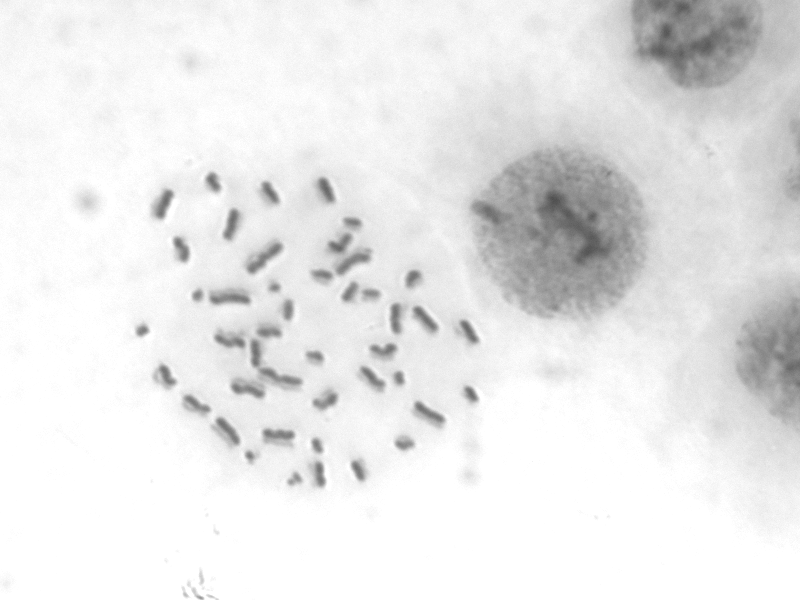

Supplement: S1 File — This file includes the underlying dataset. (ZIP) [file pone.0205766.s001.zip › fig 1D left bottom.tif]

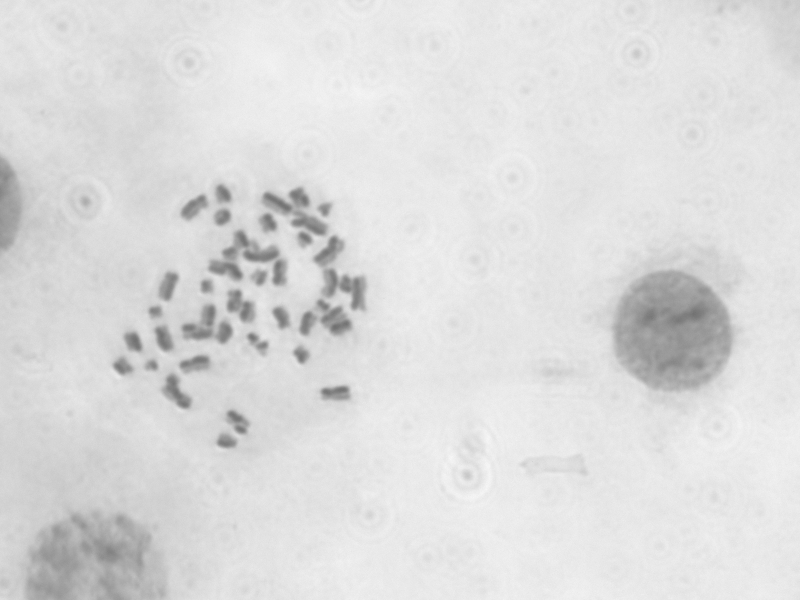

Supplement: S1 File — This file includes the underlying dataset. (ZIP) [file pone.0205766.s001.zip › fig 1D left top.tif]

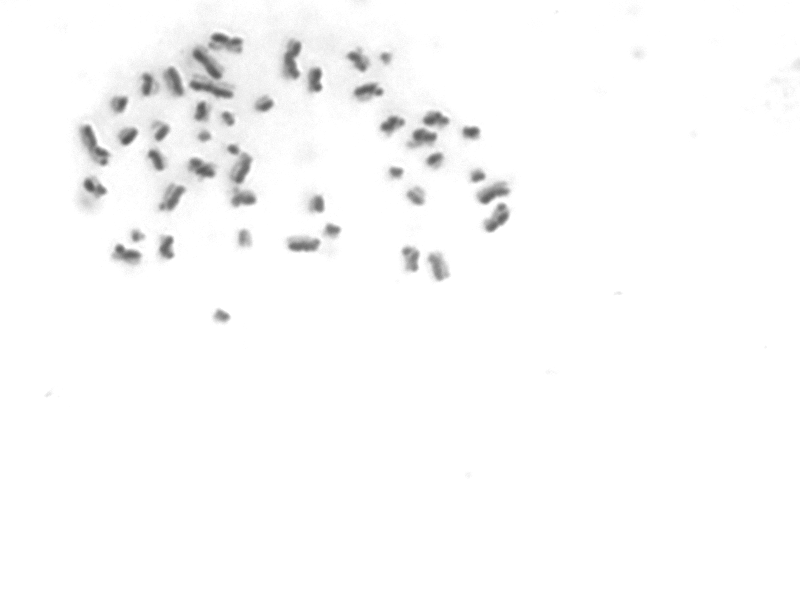

Supplement: S1 File — This file includes the underlying dataset. (ZIP) [file pone.0205766.s001.zip › fig 1D right bottom.tif]

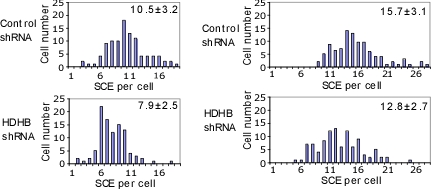

Supplement: S1 File — This file includes the underlying dataset. (ZIP) [file pone.0205766.s001.zip › fig 1E 1F.jpg]

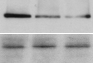

Supplement: S1 File — This file includes the underlying dataset. (ZIP) [file pone.0205766.s001.zip › fig 2B.jpg]

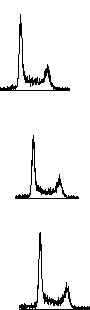

Supplement: S1 File — This file includes the underlying dataset. (ZIP) [file pone.0205766.s001.zip › fig 2C.jpg]

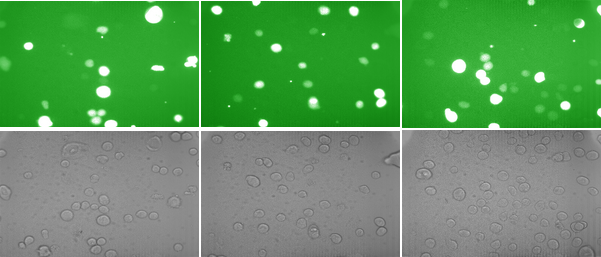

Supplement: S1 File — This file includes the underlying dataset. (ZIP) [file pone.0205766.s001.zip › fig 2D.tif]

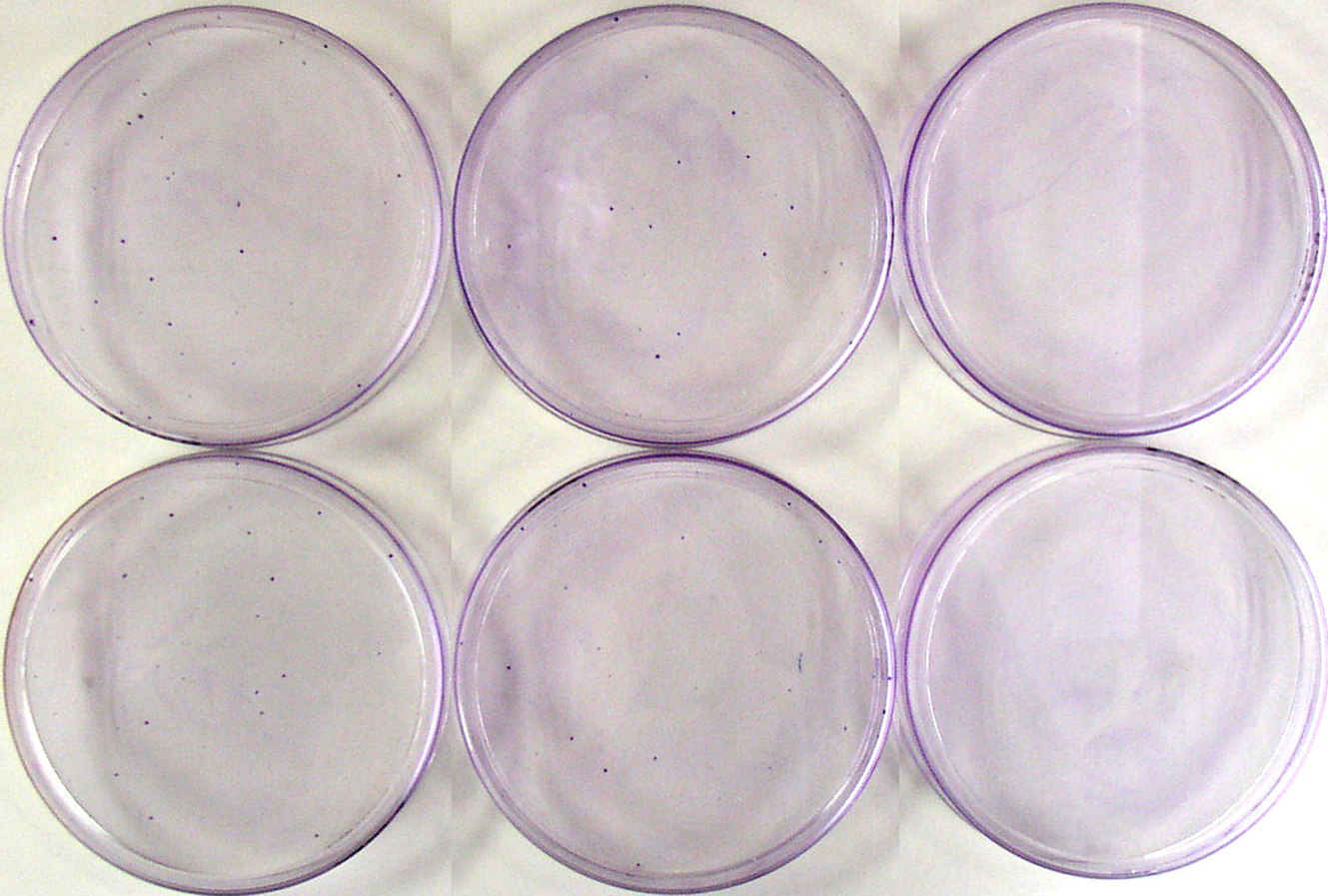

Supplement: S1 File — This file includes the underlying dataset. (ZIP) [file pone.0205766.s001.zip › Fig 2E.jpg]

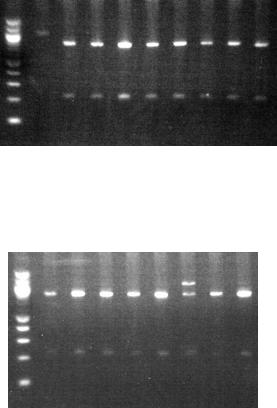

Supplement: S1 File — This file includes the underlying dataset. (ZIP) [file pone.0205766.s001.zip › fig 2G.tif]

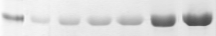

Supplement: S1 File — This file includes the underlying dataset. (ZIP) [file pone.0205766.s001.zip › Fig 2i-1.tif]

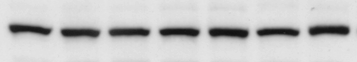

Supplement: S1 File — This file includes the underlying dataset. (ZIP) [file pone.0205766.s001.zip › fig 2i-2.tif]

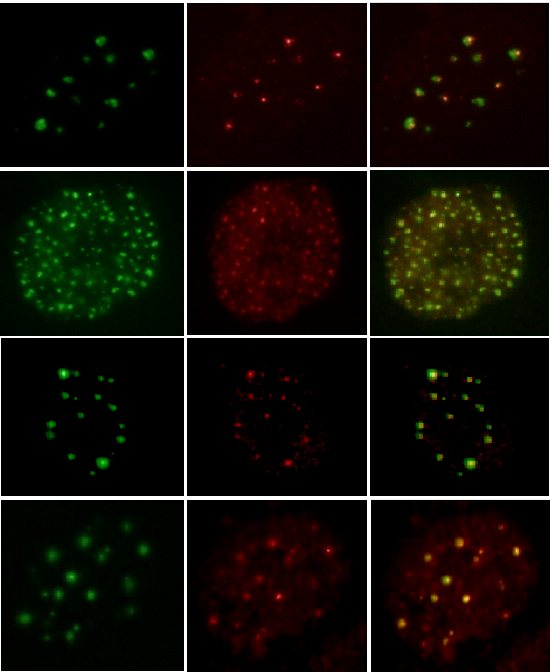

Supplement: S1 File — This file includes the underlying dataset. (ZIP) [file pone.0205766.s001.zip › fig 3A left.tif]

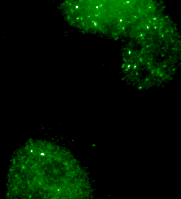

Supplement: S1 File — This file includes the underlying dataset. (ZIP) [file pone.0205766.s001.zip › fig 3A right bottom row HDHB.tif]

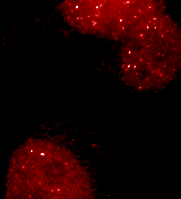

Supplement: S1 File — This file includes the underlying dataset. (ZIP) [file pone.0205766.s001.zip › fig 3A right bottom row RPA.tif]

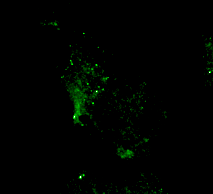

Supplement: S1 File — This file includes the underlying dataset. (ZIP) [file pone.0205766.s001.zip › fig 3A right middle row HDHB.tif]

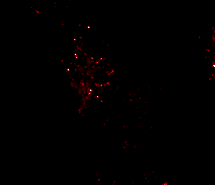

Supplement: S1 File — This file includes the underlying dataset. (ZIP) [file pone.0205766.s001.zip › fig 3A right middle row rad51.tif]

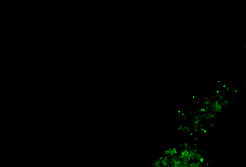

Supplement: S1 File — This file includes the underlying dataset. (ZIP) [file pone.0205766.s001.zip › fig 3A right top row HDHB.tif]

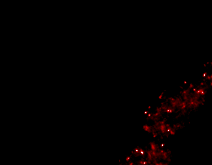

Supplement: S1 File — This file includes the underlying dataset. (ZIP) [file pone.0205766.s001.zip › fig 3A right top row Rad52.tif]

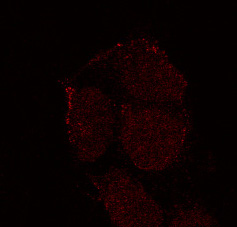

Supplement: S1 File — This file includes the underlying dataset. (ZIP) [file pone.0205766.s001.zip › Fig 3C bottom left panel image3.jpg]

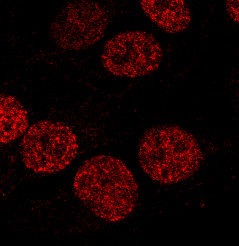

Supplement: S1 File — This file includes the underlying dataset. (ZIP) [file pone.0205766.s001.zip › Fig 3C bottom middle panel image12.jpg]

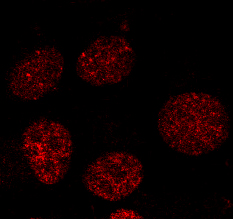

Supplement: S1 File — This file includes the underlying dataset. (ZIP) [file pone.0205766.s001.zip › Fig 3C bottom right panel image13.jpg]

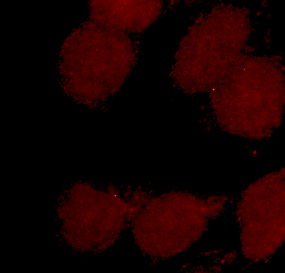

Supplement: S1 File — This file includes the underlying dataset. (ZIP) [file pone.0205766.s001.zip › Fig 3C top left panel image6.tif]

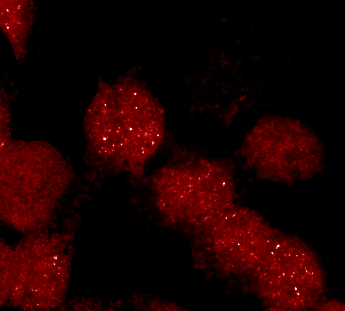

Supplement: S1 File — This file includes the underlying dataset. (ZIP) [file pone.0205766.s001.zip › Fig 3C top middle panel image 4.tif]

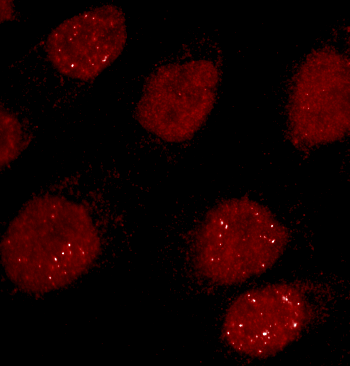

Supplement: S1 File — This file includes the underlying dataset. (ZIP) [file pone.0205766.s001.zip › Fig 3C top right panel image5.tif]

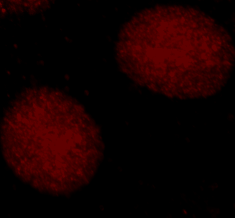

Supplement: S1 File — This file includes the underlying dataset. (ZIP) [file pone.0205766.s001.zip › Fig 3F 1st panel image8.tif]

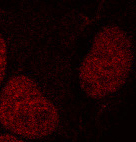

Supplement: S1 File — This file includes the underlying dataset. (ZIP) [file pone.0205766.s001.zip › Fig 3F 2nd panel image 7.jpg]

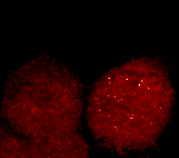

Supplement: S1 File — This file includes the underlying dataset. (ZIP) [file pone.0205766.s001.zip › Fig 3F 3rd panel image9.tif]

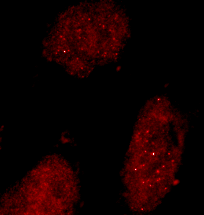

Supplement: S1 File — This file includes the underlying dataset. (ZIP) [file pone.0205766.s001.zip › Fig 3F 4th panel image10.tif]

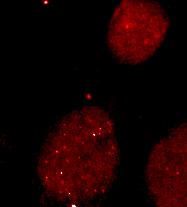

Supplement: S1 File — This file includes the underlying dataset. (ZIP) [file pone.0205766.s001.zip › Fig 3F 5th panel image11.tif]

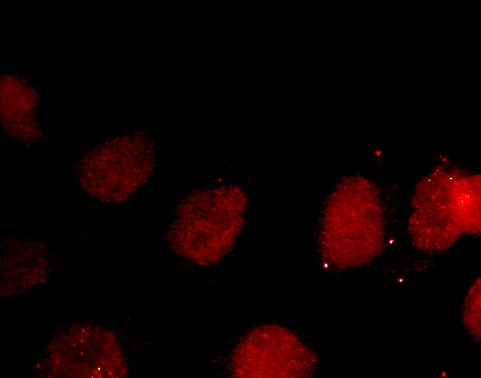

Supplement: S1 File — This file includes the underlying dataset. (ZIP) [file pone.0205766.s001.zip › fig 3G left bottom.tif]

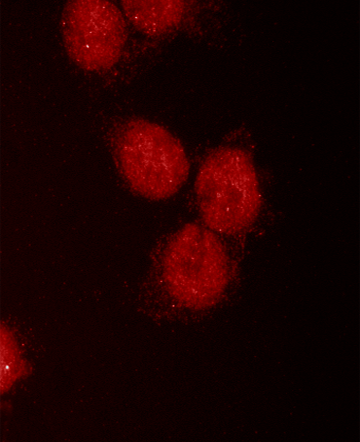

Supplement: S1 File — This file includes the underlying dataset. (ZIP) [file pone.0205766.s001.zip › fig 3G left top.tif]

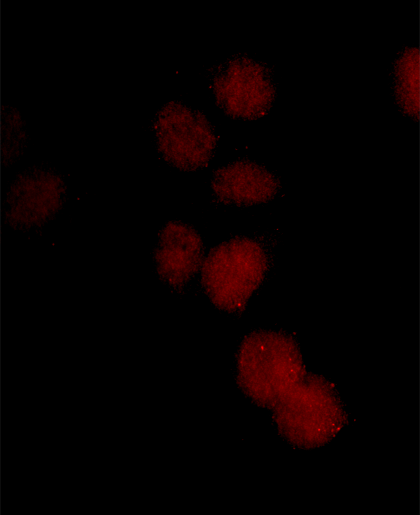

Supplement: S1 File — This file includes the underlying dataset. (ZIP) [file pone.0205766.s001.zip › fig 3G right bottom.tif]

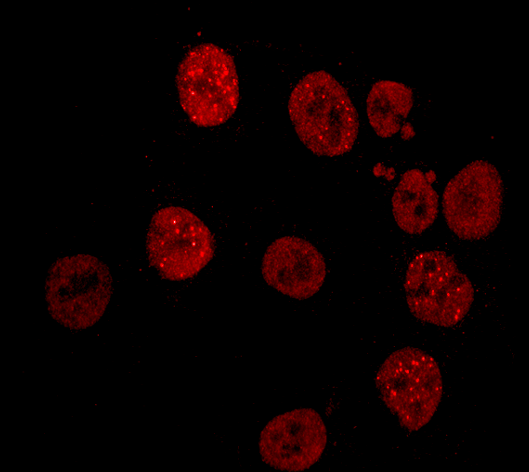

Supplement: S1 File — This file includes the underlying dataset. (ZIP) [file pone.0205766.s001.zip › fig 3G right top.tif]

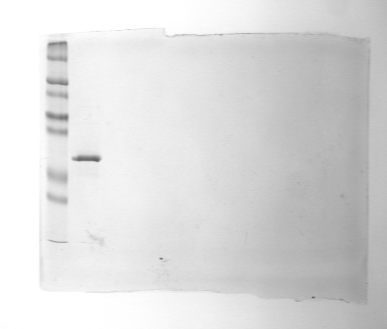

Supplement: S1 File — This file includes the underlying dataset. (ZIP) [file pone.0205766.s001.zip › fig 4B.tif]

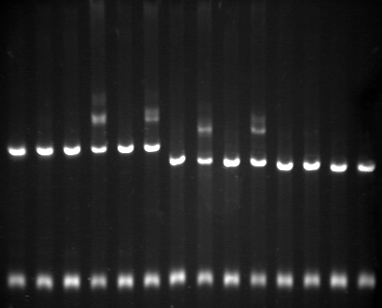

Supplement: S1 File — This file includes the underlying dataset. (ZIP) [file pone.0205766.s001.zip › Fig 4C substrates.tif]

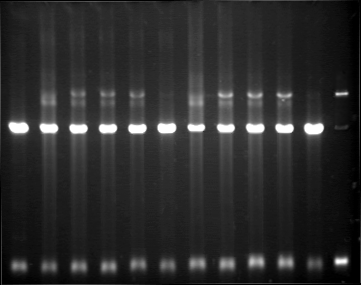

Supplement: S1 File — This file includes the underlying dataset. (ZIP) [file pone.0205766.s001.zip › Fig 4D cacl2-pst1''.tif]

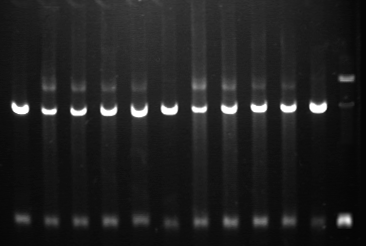

Supplement: S1 File — This file includes the underlying dataset. (ZIP) [file pone.0205766.s001.zip › Fig 4E cacl2-xho1'.tif]

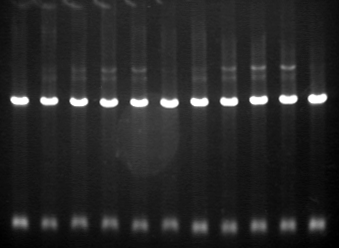

Supplement: S1 File — This file includes the underlying dataset. (ZIP) [file pone.0205766.s001.zip › Fig 4F ammo-pst1.tif]

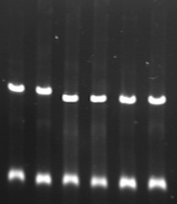

Supplement: S1 File — This file includes the underlying dataset. (ZIP) [file pone.0205766.s001.zip › Fig 4G blunt.tif]

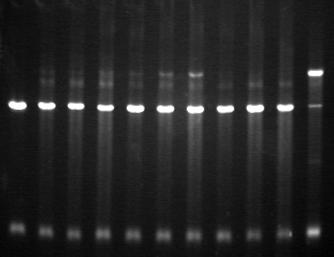

Supplement: S1 File — This file includes the underlying dataset. (ZIP) [file pone.0205766.s001.zip › Fig 4H mutB'.tif]

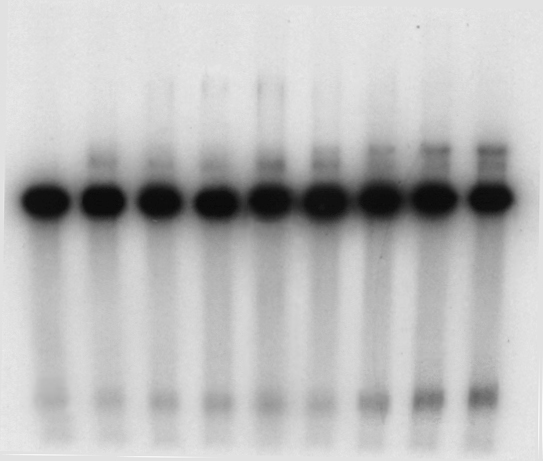

Supplement: S1 File — This file includes the underlying dataset. (ZIP) [file pone.0205766.s001.zip › Fig 5A radio.tif]

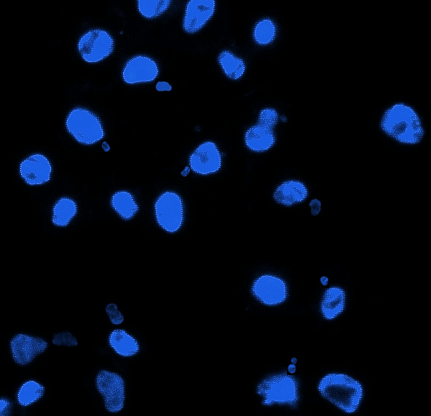

Supplement: S1 File — This file includes the underlying dataset. (ZIP) [file pone.0205766.s001.zip › Fig1B top right panel image2.tif]
